# Retrospective Longitudinal Observational Study on the Long-Term Effects of Sodium-Glucose Cotransporter-2 Inhibitors on the Development of Metabolic Dysfunction-Associated Steatotic Liver Disease in Type 2 Diabetic Japanese Patients

**DOI:** 10.3390/jcm13164929

**Published:** 2024-08-21

**Authors:** Hisayuki Katsuyama, Seiichi Horinaka, Mariko Hakoshima, Hiroki Adachi, Hidekatsu Yanai

**Affiliations:** Department of Diabetes, Endocrinology and Metabolism, National Center for Global Health and Medicine Kohnodai Hospital, 1-7-1 Kohnodai, Ichikawa 272-8516, Chiba, Japan; d-katsuyama@hospk.ncgm.go.jp (H.K.); d-22horinaka@hospk.ncgm.go.jp (S.H.); d-hakoshima@hospk.ncgm.go.jp (M.H.); dadachidm@hospk.ncgm.go.jp (H.A.)

**Keywords:** cardiovascular disease, hepatic fibrosis, metabolic dysfunction-associated steatotic liver disease, sodium-glucose cotransporter 2 inhibitors, serum lipids

## Abstract

**Background/Objectives**: The health burden of metabolic dysfunction-associated fatty liver disease (MASLD) has been increasing lately. Cardiovascular disease (CVD) is the main cause of death in MASLD patients; therefore, the treatments for MASLD should improve both CV risk factors such as obesity, diabetes, and dyslipidemia, in addition to an improvement in liver function. The evidence on the long-term effects of sodium-glucose cotransporter 2 inhibitors (SGLT2is) on the progression of MASLD in Asian populations is very limited. **Methods**: The retrospective longitudinal study was performed by using the medical records at our institute. We picked up patients with type 2 diabetes who had taken SGLT2is for at least 3 years or longer between 1 April 2014 and 31 March 2018. We collected the data on metabolic parameters, including laboratory data and anthropometric parameters, and compared the data before and after the initiation of SGLT2is treatment. **Results**: During the observation period, 324 patients had taken SGLT2is for 3 years. Three-year SGLT2is treatment significantly reduced body weight, hemoglobin A1c, low-density lipoprotein cholesterol, triglyceride, and non-high-density lipoprotein cholesterol (non-HDL-C). Such favorable changes in serum lipids were remarkable in patients with statins. Furthermore, this treatment significantly improved liver function and the markers for hepatic steatosis and hepatic fibrosis. **Conclusions**: Considering that the development of CVD determines the prognosis of MASLD patients, long-term SGLT2is treatment may be an ideal therapy for MASLD patients.

## 1. Introduction

Metabolic dysfunction-associated steatotic liver disease (MASLD), previously termed nonalcoholic fatty liver disease (NAFLD), is defined as the presence of hepatic steatosis in conjunction with at least one cardiometabolic risk factor (obesity, hypertension, type 2 diabetes, dyslipidemia) [1]. Approximately 1/4 of people with hepatic steatosis progress to metabolic dysfunction-associated steatohepatitis (MASH), which is characterized by hepatocellular ballooning and lobular necroinflammation, and an increased risk of fibrosis, cirrhosis, hepatic decompensation, hepatocellular carcinoma, and all-cause mortality [2]. The global burden of MASLD is increasing at an alarming rate, with global prevalence reaching up to 32.4% [3,4].

A retrospective analysis of patients with MASLD showed that cardiovascular disease (CVD) (38.3%) was the leading cause of death in MASLD patients [5], indicating that CVD was the most crucial determinant of mortality in MASLD patients. Considering that the development of CVD determines the prognosis of MASLD patients, the treatments for MASLD should improve cardiometabolic risk factors such as obesity and dyslipidemia, in addition to improving liver function [6]. The metabolic syndrome is significantly associated with the development of MASLD and each component of metabolic syndrome is also associated with the development of MASLD [7]. Briefly, obesity, type 2 diabetes, hypertension, and dyslipidemia such as high triglyceride (TG) and low high-density lipoprotein cholesterol (HDL-C) levels induce the development of MASLD [7]. Obesity, especially visceral obesity, is the most common and well-documented risk factor for MASLD. In the meta-analysis, the pooled odds ratios (ORs) for the formation of MASLD in waist circumference and body mass index (BMI) were 2.34 (95%CI [confidence interval], 1.83 to 3.00), and 2.85 (95%CI, 1.60 to 5.08), respectively [8].

Sodium-glucose cotransporter 2 inhibitors (SGLT2is) are reversible inhibitors of SGLT2, leading to reduction in renal glucose reabsorption and decrease in plasma glucose, in an insulin-independent manner [9,10,11]. This property of SGLT2is is beneficial to reduce visceral fat as well as body weight [11]. A recent meta-analysis provided evidence that SGLT2is significantly reduced visceral adipose tissue, subcutaneous adipose tissue, and ectopic liver fat, in addition to body weight, especially in young type 2 diabetic patients with MASLD and high BMI [12].

SGLT2is have multifactorial beneficial properties for MASLD. Liver X receptor (LXR), the potential glucose sensor, comprises two isotypes, LXRα and LXRβ [13]. They function as nuclear receptors with crucial roles in lipid metabolism [14,15,16]. LXRα is predominantly found in metabolically active tissues such as the liver [17,18]. An increase in LXR expression has been demonstrated to correlate with the worsening of MASH [19,20]. Bile acids (BAs) are well known to facilitate the absorption of dietary fat and fat-soluble molecules. The presence of metabolic disorders such as MASLD has been reported to be closely related to abnormal levels of BAs in the blood and fecal metabolites of such patients [21]. Thus, the gut microbiota interacting with BAs and altering BA metabolism are critical in the pathogenesis of such diseases. One of the SGLT2is, dapagliflozin, decreased hepatic lipid contents via inhibiting the expression of LXRα and downstream lipogenesis genes [22]. Dapagliflozin downregulated LXRα expression through increasing LXRα degradation. Furthermore, dapagliflozin reversed gut dysbiosis induced by BA disruption and altered gut microbiota profile to reduce intestinal lipids absorption [22]. 

Hepatic fibrosis is a severe pathological change in the progression of MASH and is characterized by the activation of hepatic stellate cells (HSCs) [23]. MicroRNAs (miRNAs) are small non-coding RNAs, approximately 21 nucleotides in length, which participate in disease progression [24,25]. miR-34a-5p was reported to promote the development of MASLD [26]. The inhibition of miR-34a-5p prevented HSCs activation by regulating the transforming growth factor-β (TGF-β) signaling pathway [27]. GREM2 was identified as a direct target of miR-34a-5p [27]. Empagliflozin ameliorated MASLD-associated fibrosis by downregulating miR-34a-5p and targeting GREM2 to inhibit the TGFβ pathway in HSCs [27].

SGLT2is improve obesity and diabetes, endoplasmic reticulum (ER) stress, oxidative stress, and inflammation, which is beneficial for MASLD [28]. However, the evidence on the effects of SGLT2is, especially the long-term effects, on the progression of MASLD in Asian populations with poorly controlled type 2 diabetes is very limited. Here, we investigated the long-term (>3 years) effect of SGLT2is on glucose/lipid metabolism and the markers for hepatic steatosis and hepatic fibrosis in Japanese patients with type 2 diabetes.

## 2. Materials and Methods

### 2.1. Study Population

The study protocol was approved by the Ethics Committee of the National Center for Global Health and Medicine (NCGM-S-004397), and the study was performed in accordance with the Declaration of Helsinki. 

We performed a retrospective longitudinal study by using the medical records at the National Center for Global Health and Medicine, Kohnodai Hospital, Japan. Patients with type 2 diabetes who had taken SGLT2is for at least 3 years or longer between 1 April 2014 and 31 March 2018 were picked up and included for the analysis. This study did not exclude patients who had taken lipid-lowering treatment after the first evaluation. As a part of our regular medical treatments, registered dietitians provide guidance on diet and exercise at our diabetes outpatient clinic. However, no special lifestyle modification guidance was provided for this research. Any anti-obesity treatments had not been started in overweight and obese patients. We excluded the patients who did not visit our hospital regularly. Therefore, only survivors and regular follow-ups were included in this study. 

### 2.2. Data Collection

We collected the data on metabolic parameters, including blood tests and anthropometric parameters, and compared the data before and after the initiation of SGLT2is treatment. Information on concomitant treatments was also collected from medical charts. Body weight, height, and blood pressure were measured according to the clinical standards. BMI was calculated by dividing body weight in kilograms by body height squared in meters. The measurements of serum hemoglobin A1c (HbA1c), total cholesterol (TC), triglyceride (TG), blood urea nitrogen (BUN), creatinine, and uric acid (UA) were performed using enzymatic assays. The hexokinase method was used for the evaluation of plasma glucose. Total protein (TP) and serum albumin were measured by the modified bromocresol green method. Total bilirubin (T-Bil) was measured by the vanadate oxidase method. A direct method was used for the measurements of serum low-density lipoprotein cholesterol (LDL-C) and HDL-C. Serum aspartate aminotransferase (AST), alanine aminotransferase (ALT), and γ-glutamyl transferase (GGT) were measured by using the Japan Society of Clinical Chemistry transferable method. The estimated glomerular filtration rate (eGFR) was calculated by age and serum creatinine based on the estimation equation for Japanese patients [29]. Non-HDL-C was calculated by subtracting HDL-C from TC. We used the hepatic steatosis index (HSI) as the marker for hepatic steatosis, and this index was calculated by using the following formula: 8 × (ALT/AST) + BMI + (2, if diabetes mellitus) + (2, if female) [30]. We used the AST-to-platelet ratio index (APRI) and fibrosis-4 (FIB-4) index as the markers for hepatic fibrosis. The APRI was calculated as follows: AST (IU/L)/Upper limit of the normal range of AST: 40 (IU/L)/Platelet count (109/L) × 100 [31]. The FIB-4 index was calculated as follows: (age × AST)/(platelet counts (×109/L) × (ALT)1/2 [32,33]. We did not perform any liver imaging studies and liver biopsy to evaluate hepatic steatosis and hepatic fibrosis.

### 2.3. Statistical Analysis

Comparisons between baseline data and the data after starting SGLT2is were analyzed by paired *t*-tests. Spearman’s rank correlation coefficient was used to determine the correlations between the parameters. Comparisons of frequency of patients who had taken each drug were performed by using Fisher’s exact probability test. Missing data were excluded from analyses. All data are expressed as mean ± SD, and *p* < 0.05 was considered to be statistically significant. Gender, age, changes in BMI, HbA1c, TG, and eGFR were used in a multiple regression analysis to identify important predictors of changes in HSI and APRI. We used SPSS version 29 (IBM Corp, Armonk, NY, USA) for statistical analysis.

## 3. Results

### 3.1. Characteristics of Patients Studied

#### 3.1.1. Clinical and Laboratory Characteristics of Patients Studied at Baseline

During the observation period, 324 patients had taken SGLT2is for 3 years. Clinical and laboratory characteristics of patients studied are shown in Table 1. The mean age of the patients was 58.9 years, and the mean BMI was 28.1 kg/m^2^, suggesting that a relatively large number of obese patients were included in this study. The mean value of HbA1c was 8.3% (67 mmol/mol), indicating that a relatively large number of patients with poor glucose control were included. Hematological data such as hemoglobin, hematocrit, and the count of platelets and the markers for renal function such as BUN, creatinine, and eGFR were within normal range in most patients. UA, TP, T-Bil, and albumin levels were also within normal range. The mean value of AST was the upper limit of normal range. The mean values of ALT and GGT were close to the upper limit of normal range in males and were above the normal range in females. The mean value of TG was above the upper limit of normal range.

#### 3.1.2. SGLT2is Used in Patients Studied

The SGLT2is which patients studied had taken are shown in Table 2. Six kinds of SGLT2is were used. Dapagliflozin and luseogliflozin were frequently used.

#### 3.1.3. Anti-Diabetic Drugs Which Had Been Prescribed along with SGLT2is

Anti-diabetic drugs which had been prescribed along with SGLT2is are shown in Table 3.

#### 3.1.4. Concomitant Use of Renin–Angiotensin System (RAS) Inhibitors and Statins

Concomitant use of RAS inhibitors and statins are shown in Table 4. Regarding RAS inhibitors, there was little difference in the proportion of patients using them at baseline and after 3 years. On the other hand, the proportion of patients using statins increased significantly by 11% over 3 years.

### 3.2. Changes in Metabolic Parameters by 3-Year SGLT2i Treatment

#### 3.2.1. Changes in Metabolic Parameters by 3-Year SGLT2is Treatment

Changes in metabolic parameters by 3-year SGLT2is treatment are shown in Table 5. Three-year SGLT2is treatment significantly reduced body weight by 3.4 kg. HbA1c was significantly reduced by 0.9% (10 mmol/mol). TC, LDL-C, TG, and non-HDL-C significantly decreased and HDL-C significantly increased. Furthermore, 3-year SGLT2is treatment significantly reduced AST, ALT, and GGT. 

#### 3.2.2. Differences in Changes in Serum Lipids after the Start of SGLT2is with and without Statins

Differences in changes in serum lipids after the start of SGLT2is with and without statins are shown in Table 6. HDL-C significantly increased in both patients with and without statins. Although significant reduction in TC, LDL-C, TG, and non-HDL-C were observed in patients with statins, significant decreases in such lipids were not observed in patients without statins.

### 3.3. Changes in the Markers for Hepatic Steatosis and Hepatic Fibrosis after the Start of SGLT2is

#### 3.3.1. Changes in HSI and APRI after the Start of SGLT2is

**Figure 1:**
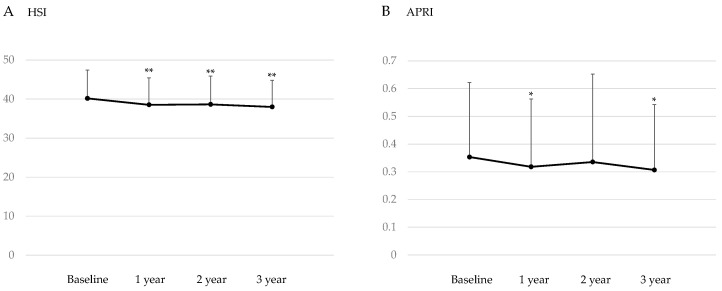
Changes in the markers for hepatic steatosis (hepatic steatosis index, HIS) (**A**) and hepatic fibrosis (AST to platelet ratio index, APRI) (**B**) after the start of SGLT2is. * *p* < 0.01 and ** *p* < 0.001 vs. baseline data.

Changes in HSI, which is the marker for hepatic steatosis, and APRI, which is the maker for hepatic fibrosis after the start of SGLT2is, are shown in Figure 1. Three-year SGLT2is treatment significantly reduced HSI, and HSI significantly decreased at one and two years after the start of SGLT2is. Three -year SGLT2is treatment also significantly reduced APRI, and APRI significantly decreased at one year after the start of SGLT2is. 

#### 3.3.2. Change in FIB-4 Index after the Start of SGLT2is

**Figure 2:**
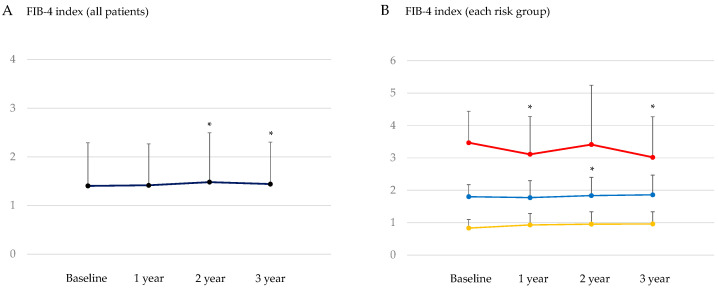
Change in the FIB-4 index after the start of SGLT2is in all patients (**A**) and in each risk group (**B**). Red, blue, and yellow lines and circles indicate high, intermediate, and low risk for advanced hepatic fibrosis, respectively. * *p* < 0.05 vs. baseline data.

Changes in the FIB-4 index, another maker for hepatic fibrosis, after the start of SGLT2is are shown in Figure 2. The analysis using all patients showed a significant increase in the FIB-4 index at 2 and 3 years after SGLT2is administration. According to FIB-4 values, patients were classified as (1) low risk for advanced hepatic fibrosis, FIB-4 < 1.3; (2) intermediate risk, FIB-4 1.3–2.66; and (3) high risk, FIB-4 ≥ 2.67 [6]. Three -year SGLT2is treatment significantly reduced the FIB-4 index in high risk patients. In such patients, the FIB-4 index significantly decreased at one year after SGLT2is administration.

#### 3.3.3. Differences in Changes in HSI and APRI after the Start of SGLT2is with and without Metformin, Pioglitazone, Glucagon-like Peptide 1 Receptor Agonists (GLP-1RAs), and Insulin

Differences in changes in HSI and APRI after the start of SGLT2is with and without metformin, pioglitazone, GLP-1RAs, and insulin are shown in Table 7. HSI significantly decreased in patients both with and without metformin. However, a significant reduction in APRI was observed only in patients with metformin. HSI significantly decreased in both patients with and without pioglitazone, and APRI did not change in both groups. HSI significantly decreased in both patients with and without GLP-1RAs. However, a significant reduction in APRI was observed only in patients without GLP-1RAs. Interestingly, any changes in HSI and APRI by 3-year SGLT2i treatment were not obtained in patients with insulin. However, 3-year SGLT2is treatment significantly reduced both HSI and APRI in patients without insulin.

#### 3.3.4. Differences in Changes in HSI and APRI after the Start of SGLT2is with and without RAS Inhibitors and Statins

Differences in changes in HSI and APRI after the start of SGLT2is with and without RAS inhibitors and statins are shown in Table 8. HSI significantly decreased in both patients with and without RAS inhibitors. However, a significant reduction in APRI was observed only in patients without RAS inhibitors. HSI significantly decreased in both patients with and without statins. However, a significant reduction in APRI was observed only in patients without statins.

### 3.4. Correlations between Changes in Metabolic Parameters by 3-Year SGLT2is Treatment

#### 3.4.1. Correlation of Changes in Serum Lipids with Changes in Metabolic Parameters at 3 Years after the Start of SGLT2is

Correlation of changes in serum lipids with changes in metabolic parameters at 3 years after the start of SGLT2is are shown in Table 9. Change in serum TG was significantly and positively correlated with changes in HbA1c, ALT, and GGT. Change in serum non-HDL-C was significantly and positively correlated with changes in HbA1c and GGT. 

#### 3.4.2. Correlation of Changes in the Markers for Hepatic Steatosis and Hepatic Fibrosis with Changes in Metabolic Parameters by 3-Year SGLT2is Treatment

Correlation of changes in the markers for hepatic steatosis and hepatic fibrosis with changes in metabolic parameters by 3-year SGLT2is treatment are shown in Table 10. The change in HSI was siginificantly and positively correlated with changes in body weight, BMI and HbA1c, and also significantly and negatively correlated with HDL-C. The change in APRI was siginificantly and positively correlated with changes in body weight, BMI, and HbA1c. The change in APRI was not correlated with changes in any serum lipids.

#### 3.4.3. Multiple Regression Analysis

Table 11a shows the variables independently associated with Δ HSI in multiple regression analysis. Δ BMI and Δ HbA1c were a significant determinant of Δ HSI. Table 11b shows the variables independently associated with Δ APRI in multiple regression analysis. A significant determinant of Δ APRI was not detected (*p* = 0.172).

## 4. Discussion

The present study demonstrates that 3-year SGLT2i treatment significantly reduced TC and LDL-C, which disagrees with the results of previous meta-analyses [34,35,36,37]. All meta-analyses showed that SGLT2is significantly increased LDL-C, and three of four meta-analyses showed a significant increase in TC by SGLT2is. Even in patients without statins, significant increases in TC and LDL-C were not observed in our study. The inclusion of a relatively large number of short-term RCTs and patients of different races in such meta-analyses can explain a different result between ours and meta-analyses. Our previous study showed that SGLT2is did not show a significant effect on LDL-C and TG at 1, 2, 3, and 6 months after the start of SGLT2is [38,39], suggesting that a long-term treatment is required to evaluate the effects of SGLT2is on serum lipids. However, a significant reduction in LDL-C and TC in our study might have been induced by the concomitant use of statins.

Our study showed that SGLT2is significantly increased HDL-C, which was observed in all meta-analyses [34,35,36,37]. A significant increase in HDL-C was observed in both patients with and without statins, suggesting that an elevation in HDL can be caused by SGLT2is alone. We also showed that SGLT2is significantly reduced TG, which was observed in three of four meta-analyses [35,36,37]. Epidemiologic and clinical trials have shown that elevated TG and low levels of HDL-C are independent risk factors for coronary heart disease. Therefore, the 2001 National Cholesterol Education Program Adult Treatment Panel III (ATP III) guidelines placed more emphasis on the importance of managing high TG and low HDL-C by identifying non-HDL-C (LDL-C + very low-density lipoprotein (VLDL)-C) [40]. An effect of SGLT2is on non-HDL-C has not been sufficiently studied, and only the present study and one meta-analysis reported an effect of SGLT2is on non-HDL-C. The meta-analysis showed that SGLT2is had a significant increase in non-HDL-C by 0.09 mmol/L (95% CI, 0.06 to 0.12; *p* < 0.00001) [37], which disagrees with our result that SGLT2is significantly reduced non-HDL-C. This difference may be due to the inclusion of a relatively large number of short-term RCTs and patients of different races in the meta-analysis. Most studies had shown that SGLT2is reduced TG [35,36,37], and TG is included in non-HDL-C. SGLT2is reduce TG, by reducing TG-rich lipoprotein which is included in non-HDL-C. Therefore, our result may be more reasonable. However, a significant decrease in TG and non-HDL-C were not observed in patients without statins. The effect observed in the study on lipid profiles is likely to be due to statin use rather than SGLT2is. Imada, T. et al. investigated the effect of combination of SGLT2is with statins by a retrospective analysis [41]. Multivariate regression analyses showed that the change in LDL-C by SGLT2is depended on statin use. Such a synergistic effect with statins was more characteristic for SGLT2is. Three-year SGLT2is treatment significantly reduced body weight and HbA1c, suggesting that long-term SGLT2is treatment improved insulin resistance. Beneficial changes in serum lipids may be induced by an improvement in insulin resistance by 3-year SGLT2is treatment. Changes in TG and non-HDL-C were significantly and positively correlated with changes in HbA1c, supporting our hypothesis. 

SGLT2is decrease blood glucose not by increasing insulin secretion, but by reducing renal glucose reabsorption. Such change in glucose and insulin relatively increases the secretion of glucagon, activating hormone-sensitive lipase (HSL) in adipose tissue [42]. Accumulated TG is hydrolyzed to free fatty acids (FFAs) by activated HSL in adipose tissue, which reduces adipose tissue size, resulting in an improvement in insulin resistance due to reduced inflammatory cytokines and increased adiponectin [43]. FFAs released from adipose tissue may be promptly used by skeletal muscles and liver because SGLT2is shift the energy metabolism towards FA utilization by the alteration of the glucose–FA cycle [44]. An increase in adiponectin levels has beneficial effects on glucose and lipid metabolism by activation of adenosine monophosphate-activated protein kinase (AMPK) [45]. SGLT2is have been reported to activate AMPK and inactivate acetyl-CoA carboxylase (ACC) which regulates FA synthesis [46]. SGLT2is reduce FA accumulation in the liver, reducing hepatic TG re-synthesis from FA. VLDL, the main component of non-HDL-C other than LDL, is produced in the liver. High TG observed in insulin resistance is induced by hepatic over-production of VLDL and reduced VLDL degradation by impaired lipoprotein lipase [47]. High VLDL causes an increase in LDL as the metabolite. VLDL is composed of cholesterol, re-synthesized TG, and apoproteins [47]. Statins reduce hepatic cholesterol synthesis and SGLT2is decrease hepatic TG synthesis, reducing VLDL production in the liver, which may contribute to an improvement in TG, LDL-C, and non-HDL-C. 

The evidence on effects of SGLT2is on MASLD is very limited. In our previous study, SGLT2is significantly improved serum transaminase at 3 and 6 months after SGLT2is initiation in type 2 diabetic patients [38,39]. We also discovered that the FIB-4 index was significantly improved by the 12-month SGLT2is treatment in a high-risk group [48]. We also reported that the FIB-4 index significantly decreased in the SGLT2is group but not in the pioglitazone group at 96 weeks [49]. However, APRI significantly decreased in both groups. SGLT2is reduced body weight by 3.2 kg; however, pioglitazone increased body weight by 1.7 kg.

Our study showed that 3-year SGLT2is treatment significantly reduced HSI as the marker for hepatic steatosis and APRI as the marker for hepatic fibrosis, and a significant reduction in the FIB-4 index in high-risk group was also observed. Changes in both HSI and APRI were significantly and positively correlated with changes in body weight, BMI, and HbA1c, suggesting that weight loss and an improvement of insulin resistance by 3-year SGLT2is treatment may improve hepatic steatosis and fibrosis. Change in HSI was significantly and negatively correlated with change in HDL-C and change in GGT was significantly and positively correlated with changes in TG and non-HDL-C, suggesting that an improvement in serum lipids by SGLT2is may also favorably influence on the progression of MASLD. The multiple regression analysis to detect a determinant of change in HSI showed that changes in BMI and HbA1c were a significant determinant of reduction in HSI. However, a significant determinant of reduction APRI was not detected. This suggests that 3-year SGLT2is treatment is more strongly associated with an improvement in hepatic steatosis.

In the study (*n* = 32) by Ito et al., the liver-to-spleen attenuation ratio increased at 5 years after the start of ipragliflozin (an SGLT2is) [50]. Ipragliflozin significantly improved serum aminotransferase and HbA1c over 5 years. Significant reductions in body weight and visceral fat area were sustained throughout the 5 years. Ipragliflozin significantly reduced serum ferritin and the FIB-4 index as the markers for hepatic fibrosis. Six Japanese patients with MASLD and type 2 diabetes were treated for the long term with canagliflozin [51]. GLP-1RAs were added to two patients after 3 years due to histological worsening. Histological improvement was obtained in half of the patients at 5 years. An improvement in the scores for steatosis, lobular inflammation, ballooning, and fibrosis stage was observed in 67% of patients at 5 years. Homeostasis model assessment of insulin resistance (HOMA-IR) and serum ferritin significantly decreased at 5 years. One-hundred nine patients who had taken SGLT2is for more than 3 years were investigated [52]. Improvements in body weight, liver transaminases, and the FIB-4 index were obtained at 3 years of SGLT2is treatment. In the intermediate-risk and high-risk groups (≥1.3 FIB-4 index), the FIB-4 index significantly decreased. 

MASLD is strongly associated with obesity, insulin resistance/hyperinsulinemia, type 2 diabetes, and dyslipidemia [53]. Therefore, SGLT2is and GLP-1RAs which have weight-losing effects, and metformin and pioglitazone which have the effect of improving insulin resistance, have been suggested to be promising therapeutic drugs for MASLD. In this study, 3-year SGLT2i treatment did not improve HSI and APRI in insulin-treated patients; however, SGLT2is significantly reduced both HSI and APRI in insulin-naïve patients, suggesting that exogeneous insulin as well as endogenous hyperinsulinemia may adversely affect MASDL and may attenuate the beneficial effects of SGLT2is for MASLD.

GLP-1RAs hold substantial therapeutic promise for managing diabetes, MASLD, and obesity [54,55,56,57]. Their mechanisms include augmenting insulin secretion, suppressing glucagon release, and modulating appetite and peripheral insulin sensitivity. The systematic review and meta-analysis using 13 randomized controlled trials (RCTs) which evaluated the impact of GLP-1RAs on visceral adipose tissue and subcutaneous adipose tissue in individuals with diabetes and MASLD or obesity showed that GLP-1RA treatment significantly reduced visceral adipose tissue (−0.55; 95% CI, −0.90 to −0.19), subcutaneous adipose tissue (−0.59; 95% CI, −0.99 to −0.19), body weight (−1.07; 95% CI, −1.67 to −0.47), and BMI (−1.10; 95% CI, −1.74 to −0.47) compared to controls [58]. GLP-1RA treatment improved fasting blood glucose, postprandial glucose, HbA1c, HOMA-IR, and FIB-4. In another systematic review and meta-analysis of 16 RCTs that used liraglutide, exenatide, dulaglutide, or semaglutide in the treatment of MASDL or MASH, as measured by liver biopsy or imaging techniques, the effect of GLP-1RAs on histologic resolution of MASH with no worsening of liver fibrosis (4.08; 95%CI, 2.54 to 6.56, *p* < 0.00001) was statistically significant [59]. In our study, 3-year SGLT2is treatment significantly improved HSI and APRI in patients who were not treated by GLP-1RAs, suggesting that long-term SGLT2is treatments improve hepatic steatosis and fibrosis as well as GLP-1RAs. 

Peroxisome proliferator-activated receptor gamma (PPARγ) agonists are insulin-sensitizing agents and improve lipid metabolism, and are widely investigated in MASDL. In the PIVENS trial [60], pioglitazone significantly reduced hepatic steatosis, lobular inflammation, and hepatic enzymes as compared with vitamin E and placebo for 96 weeks in patients with MASH. A meta-analysis showed that pioglitazone significantly improves liver fibrosis as compared with placebo in MASLD patients [61]. Pioglitazone is recommended in persons with type 2 diabetes and biopsy-proven MASH by the American Association of Clinical Endocrinologists [62]. Long-term pioglitazone treatment significantly improved liver-to-spleen attenuation ratio and serum aminotransferase [50]. However, the use of PPARγ agonists is associated with poor bone quality, edema, and weight gain [63]. In this study, there was no difference in the effects of 3-year SGLT2is treatment on HSI and APRI with or without the concomitant use of pioglitazone.

Metformin can activate AMPK by inhibiting mitochondrial complex 1, and activated AMPK can reduce FA synthesis [64]. In addition, metformin can reduce reactive oxygen species production by inhibiting mitochondrial complex 1, which can reduce liver damage. Therefore, metformin has been found to alleviate MASLD [64]. The meta-analysis including 10 RCTs with 459 patients showed that, compared to the controls, metformin could effectively reduce the serum fasting glucose and insulin levels and the HOMA-IR in MASLD patients at the 6-month follow-up and ALT and the HOMA-IR index at the 12-month follow-up [65]. Another meta-analysis showed that metformin decreased ALT by −10.84 (95% CI, −21.85 to 0.16; *p* = 0.05), AST by −4.82 (95% CI, −9.33 to −0.30; *p* = 0.04), and HOMA-IR by −0.42 (95% CI, −0.82 to −0.02; *p* = 0.04) in MASLD patients [66]. In our study, significant reductions in both HSI and APRI were observed in patients treated with a combination of SGLT2is and metformin, and APRI was not reduced in patients without metformin, indicating that the combination of SGLT2is with metformin has a synergistic effect on improving MASLD. The reports on a synergistic effect of the combination of SGLT2is and metformin on improving MASLD are very limited. There are only two reports, and one using mice, showing the combination therapy additively improved MASH as compared with SGLT2i or metformin alone [67]. In patients with type 2 diabetes, the combination of empagliflozin and metformin ameliorated liver steatosis, ALT levels, body weight, and glycated hemoglobin after a 6-month follow-up, as compared with the metformin monotherapy [68]. Both SGLT2is and metformin activate AMPK and inactivate ACC, reducing hepatic FA synthesis and increasing FA oxidation which results in a decrease in hepatic FA accumulation. The combination treatment may potentiate such a cascade which is beneficial for the development of MASLD. However, to prove this hypothesis, further studies should be performed in the future.

We have to mention the limitations of our study. All subjects in this study were Japanese, and the results obtained in our study may not necessarily be obtained in other ethnicities. There was no liver imaging and histological examination to prove the existence of hepatic steatosis and hepatic fibrosis. The patients’ lifestyle changes such as diet and exercise might have influenced our result, which was not investigated. We investigated differences in changes in HSI and APRI after the start of SGLT2is with and without metformin, pioglitazone, GLP-1RAs, insulin, RAS inhibitors, and statins. However, the study did not take into account the effects of the possibility that the patients studied had taken multiple classes of drugs at the same time. Our patients had poor glycemic control; therefore, SGLT2is may have shown a significant improvement in MASLD. 

## 5. Conclusions

Long-term SGLT2is treatment significantly reduced atherogenic lipids such as LDL-C, TG, and non-HDL-C which was remarkable in patients with statins, and this treatment increased HDL. Furthermore, this treatment improved hepatic steatosis and hepatic fibrosis. Considering that the development of CVD determines the prognosis of MASLD patients, long-term SGLT2i treatment may be an ideal therapy for MASLD patients.

## Figures and Tables

**Table 1:** Clinical and laboratory characteristics of patients studied at baseline (*n* = 324).

Clinical Characteristics		
Gender (male/female)	188/136	
Age (years)	58.9 ± 14.2	
Body height (cm)	163.0 ± 10.0	
Body weight (kg)	74.3 ± 18.2	
Body mass index (kg/m^2^)	28.1 ± 5.9	
Patients with body mass index ≧ 25 kg/m^2^ (*n*, %)	104, 32.1%	
Systolic blood pressure (mmHg)	133.3 ± 17.9	
Diastolic blood pressure (mmHg)	76.8 ± 12.1	
Laboratory characteristics		
	Data at baseline	Normal range
Plasma glucose (mg/dL)	192 ± 83	<110
Hemoglobin A1c (%)	8.3 ± 1.7	4.9–6.0
HbA1c (mmol/mol)	67 ± 14	30–42
Patients with HbA1c < 6.5% (*n*, %)	26, 8.0%	
Hemoglobin (g/dL)	13.9 ± 1.8	Male 13.7–16.8 Female 11.6–14.8
Hematocrit (%)	41.9 ± 5.0	Male 40.7–50.1 Female 35.1–44.4
Platelets (×10^4^/mL)	24.0 ± 7.5	158–348
Blood urea nitrogen (mg/dL)	16.5 ± 6.2	8~20
Creatinine (mg/dL)	0.8 ± 0.3	Male 0.65–1.07 Female 0.46–0.79
eGFR (mL/min/1.73 m^2^)	78 ± 28	60<
UA (mg/dL)	5.5 ± 1.4	<7
TP (g/dL)	7.2 ± 0.5	6.6–8.1
Albumin (g/dL)	4.2 ± 0.5	4.1–5.1
T-Bil	0.7 ± 0.3	0.4–1.5
AST (IU/L)	30 ± 20	13–30
ALT (IU/L)	38 ± 32	Male 10–42 Female 7–23
GGT (IU/L)	58 ± 69	Male 13–64 Female 9–32
TC (mg/dL)	187 ± 40	<220
HDL-C (mg/dL)	50 ± 13	<40
LDL-C (mg/dL)	105 ± 32	<140
TG (mg/dL)	185 ± 126	Non-fasting value < 175
Non-HDL-C (mg/dL)	138 ± 39	<170

Presented values indicate mean ± SD. ALT, alanine aminotransferase; AST, aspartate aminotransferase; GGT, gamma-glutamyl transferase; eGFR, estimated glomerular filtration rate; HDL-C, high-density lipoprotein cholesterol; LDL-C, low-density lipoprotein cholesterol; Non-HDL-C, non-high-density lipoprotein cholesterol; T-Bil, total bilirubin; TC, total cholesterol; TG, triglyceride; TP, total protein; UA, uric acid.

**Table 2:** SGLT2is which patients studied had taken.

Kind of SGLT2is	*n* (%)
Dapagliflozin	98 (30%)
Luseogliflozin	96 (30%)
Tofogliflozin	36 (11%)
Ipragliflozin	34 (10%)
Canagliflozin	34 (10%)
Empagliflozin	30 (9%)

**Table 3:** Anti-diabetic drugs which had been prescribed along with SGLT2is.

	Baseline	After 3 Years	*p* Values
Metformin	175 (54%)	198 (61%)	0.068
Pioglitazone	85 (26%)	105 (32%)	0.084
GLP-1RAs	31 (10%)	75 (23%)	<0.001
Insulin	60 (19%)	44 (14%)	0.087

GLP-1RAs, glucagon-like peptide 1 receptor agonists.

**Table 4:** Concomitant use of renin–angiotensin system (RAS) inhibitors and statins.

	Baseline	After 3 Years	*p* Values
RAS inhibitors	146 (45%)	152 (47%)	0.694
Statins	161 (50%)	197 (61%)	0.006

RAS, renin–angiotensin system.

**Table 5:** Changes in metabolic parameters by 3-year SGLT2is treatment.

	Baseline	After 3 Years	*p* Values
Body weight (kg)	74.3 ± 18.2	70.9 ± 17.6	<0.001
Body mass index (kg/m^2^)	28.1 ± 5.9	26.8 ± 5.5	<0.001
Plasma glucose (mg/dL)	192 ± 83	162 ± 69	<0.001
HbA1c (%)	8.3 ± 1.7	7.4 ± 1.2	<0.001
HbA1c (mmol/mol)	67 ± 14	57 ± 10	<0.001
TC (mg/dL)	187 ± 40	177 ± 33	<0.001
HDL-C (mg/dL)	50 ± 13	53 ± 14	<0.001
LDL-C (mg/dL)	105 ± 32	98 ± 27	0.023
TG (mg/dL)	185 ± 126	161 ± 103	0.001
Non-HDL-C (mg/dL)	138 ± 39	124 ± 32	<0.001
AST (IU/L)	30 ± 20	26 ± 17	0.004
ALT (IU/L)	38 ± 32	31 ± 32	0.009
GGT (IU/L)	58 ± 69	53 ± 101	0.014

Presented values indicate mean ± SD. ALT, alanine aminotransferase; AST, aspartate aminotransferase; GGT, gamma-glutamyl transferase; HDL-C, high-density lipoprotein cholesterol; LDL-C, low-density lipoprotein cholesterol; Non-HDL-C, non-high-density lipoprotein cholesterol; TC, total cholesterol; TG, triglyceride.

**Table 6:** Changes in serum lipids by 3-year SGLT2is treatment with and without statins.

Serum Lipids	Baseline	After 3 Years	*p* Values	Baseline	After 3 Years	*p* Values
	with Statins (*n* = 209)			without Statins (*n* = 105)		
TC	183 ± 41	167 ± 28	<0.001	195 ± 37	194 ± 33	0.868
HDL-C	50 ± 12	53 ± 14	<0.001	49 ± 13	53 ± 15	<0.001
LDL-C	102 ± 33	92 ± 26	0.003	110 ± 29	111 ± 24	0.502
TG	182 ± 119	155 ± 89	0.002	191 ± 139	173 ± 124	0.298
Non-HDL-C	133 ± 40	114 ± 25	<0.001	146 ± 36	141 ± 35	0.291

Presented values indicate mean ± SD. HDL-C, high-density lipoprotein cholesterol; LDL-C, low-density lipoprotein cholesterol; Non-HDL-C, non-high-density lipoprotein cholesterol; TC, total cholesterol; TG, triglyceride.

**Table 7:** Changes in metabolic parameters by 3-year SGLT2i treatment with and without metformin, pioglitazone, GLP-1RAs, and insulin.

	Baseline	After 3 Years	*p* Values	Baseline	After 3 Years	*p* Values
	with Metformin (*n* = 212)			without Metformin (*n* = 112)		
HSI	41.3 ± 7.4	38.6 ± 6.8	<0.001	38.1 ± 6.5	36.8 ± 6.6	0.013
APRI	0.338 ± 0.262	0.277 ± 0.208	0.001	0.383 ± 0.278	0.364 ± 0.274	0.678
	With pioglitazone (*n* = 117)			Without pioglitazone (*n* = 207)		
HSI	40.1 ± 6.2	37.6 ± 6.1	<0.001	40.2 ± 7.7	38.2 ± 7.1	<0.001
APRI	0.329 ± 0.236	0.296 ± 0.231	0.084	0.367 ± 0.284	0.313 ± 0.228	0.052
	With GLP-1RAs (*n* = 76)			Without GLP-1RAs (*n* = 248)		
HSI	42.1 ± 7.3	39.4 ± 6.8	0.002	39.5 ± 7.1	37.5 ± 6.7	<0.001
APRI	0.366 ± 0.266	0.312 ± 0.226	0.112	0.349 ± 0.269	0.304 ± 0.239	0.039
	With insulin (*n* = 74)			Without insulin (*n* = 250)		
HSI	41.2 ± 6.0	39.6 ± 5.3	0.088	39.9 ± 7.5	37.6 ± 7.1	< 0.001
APRI	0.335 ± 0.292	0.282 ± 0.199	0.476	0.359 ± 0.261	0.314 ± 0.245	0.012

Presented values indicate mean ± SD. APRI, AST-to-platelet ratio index; HIS, hepatic steatosis index; GLP-1RAs, glucagon-like peptide 1 receptor agonists.

**Table 8:** Changes in metabolic parameters by 3-year SGLT2i treatments with and without RAS inhibitors and statins.

	Baseline	After 3 Years	*p* Values	Baseline	After 3 Years	*p* Values
	RAS Inhibitors (*n* = 176)			without RAS Inhibitors (*n* = 148)		
HSI	40.1 ± 6.7	38.3 ± 6.6	<0.001	40.4 ± 8.0	37.5 ± 7.0	<0.001
APRI	0.339 ± 0.275	0.301 ± 0.247	0.142	0.370 ± 0.259	0.313 ± 0.220	0.028
	With statins (*n* = 209)			Without statins (*n* = 105)		
HSI	39.9 ± 6.7	37.4 ± 6.1	<0.001	40.7 ± 8.2	39.0 ± 7.9	<0.001
APRI	0.347 ± 0.278	0.313 ± 0.257	0.216	0.365 ± 0.250	0.295 ± 0.191	0.003
APRI	0.335 ± 0.292	0.282 ± 0.199	0.476	0.359 ± 0.261	0.314 ± 0.245	0.012

Presented values indicate mean ± SD. APRI, AST-to-platelet ratio index; HIS, hepatic steatosis index; RAS, renin–angiotensin system.

**Table 9:** Correlation of changes in serum lipids with changes in metabolic parameters at 3 years after the start of SGLT2is.

	Δ HDL-C	Δ LDL-C	Δ TG	Δ Non-HDL-C
Δ Body weight	−0.073	−0.099	−0.032	−0.029
Δ BMI	−0.098	−0.152	−0.022	−0.013
Δ HbA1c	0.033	0.033	0.165 **	0.151 *
Δ AST	0.079	0.079	0.093	0.025
Δ ALT	−0.044	0.037	0.119 *	−0.011
Δ GGT	−0.021	0.119	0.285 **	0.217 **

Presented values indicate correlation coefficients. * *p* < 0.05, ** *p* < 0.01. Δ indicate the changes in each parameter at 3 years after the start of SGLT2is. BMI, body mass index; GGT, gamma-glutamyl transferase; HDL-C, high-density lipoprotein cholesterol; LDL-C, low-density lipoprotein cholesterol; Non-HDL-C, non-high-density lipoprotein cholesterol; TG, triglyceride.

**Table 10:** Correlation of changes in the markers for hepatic steatosis (HSI) and hepatic fibrosis (APRI) with changes in metabolic parameters at 3 years after the start of SGLT2is.

	Δ HSI	Δ APRI
Δ Body weight	0.583 **	0.273 **
Δ BMI	0.593 **	0.258 **
Δ HbA1c	0.291 **	0.143 *
Δ TG	0.007	0.054
Δ HDL-C	−0.228 **	0.063
Δ LDL-C	−0.121	0.063
Δ Non-HDL-C	−0.086	−0.049

Presented values indicate correlation coefficients. * *p* < 0.05, ** *p* < 0.01. APRI, AST to platelet relation index; BMI, body mass index; HDL-C, high-density lipoprotein cholesterol; HIS, hepatic steatosis index; LDL-C, low-density lipoprotein cholesterol; Non-HDL-C, non-high-density lipoprotein cholesterol; TG, triglyceride.

**Table 11:** Multiple regression analysis with (**a**) Δ HSI and (**b**) Δ APRI as the dependent variable.

(a) Multiple Regression Analysis with Δ HSI as the Dependent Variable (*p* < 0.01)				
Independent Variables	B	SE	Standardized β	*p* Values
Gender	0.211	0.486	0.032	0.665
Age	0.010	0.018	0.043	0.559
Δ BMI	0.882	0.120	0.554	<0.001
Δ HbA1c	0.312	0.147	0.161	0.036
Δ TG	−0.001	0.002	−0.037	0.628
Δ eGFR	0.001	0.018	0.003	0.965
**(b)** **Multiple regression analysis with** **Δ APRI as the dependent variable (*p* = 0.172)**				
**Independent variables**	**B**	**SE**	**Standardized β**	***p*** **values**
Gender	−0.044	0.035	−0.112	0.207
Age	0.001	0.001	0.068	0.439
Δ BMI	0.019	0.009	0.203	0.025
Δ HbA1c	0.002	0.010	0.020	0.826
Δ TG	−0.000	0.000	−0.094	0.296
Δ eGFR	0.001	0.001	0.045	0.615

B, unstandardized beta; BMI, body mass index; eGFR, estimated glomerular filtration rate; HbA1c, hemoglobin A1c; SE, standard error for unstandardized beta; TG, triglyceride.

## Data Availability

The data supporting the findings of this study are available from the corresponding author upon reasonable request.
